# Metacognition, social cognition, and mentalizing in psychosis: are these distinct constructs when it comes to subjective experience or are we just splitting hairs?

**DOI:** 10.1186/s12888-021-03338-4

**Published:** 2021-07-02

**Authors:** P. H. Lysaker, S. Cheli, G. Dimaggio, B. Buck, K. A. Bonfils, K. Huling, C. Wiesepape, J. T. Lysaker

**Affiliations:** 1 Richard L Roudebush VA Medical Center, Department of Psychiatry, 1481 W. 10th St., Indianapolis, IN 46202 USA; 2 Department of Psychiatry, Indiana University School of Medicine, 340 W. 10th St., Indianapolis, IN 46202 USA; 3 University of Florence, School of Human Health Sciences, Piazza di San Marco, 4, 50121 Florence, FI Italy; 4 Terzocentro di Psicoterapia Cognitiva, Associazione di Psicologia Cognitiva, Via Ravenna, 9, 00161 Rome, RM Italy; 5 Department of Psychiatry and Behavioral Sciences, University of Washington, Behavioral Research in Technology and Engineering (BRiTE) Center, 1851 NE Grant Ln., Seattle, WA 98185 USA; 6 University of Southern Mississippi, School of Psychology, 118 College Dr., Hattiesbury, MS 39406 USA; 7 University of Indianapolis, School of Psychological Sciences, 1400 E. Hanna Ave., Indianapolis, IN 46277 USA; 8 Indiana State University, Department of Psychology, 200 N. 7th St., Terre Haute, IN 47809 USA; 9 Department of Philosophy, Emory University, 201 Dowman Dr., Atlanta, GA 30322 USA

**Keywords:** Metacognition, Mentalizing, Social cognition, Self, Psychosis, Schizophrenia, Recovery, Rehabilitation, Psychotherapy

## Abstract

Research using the integrated model of metacognition has suggested that the construct of metacognition could quantify the spectrum of activities that, if impaired, might cause many of the subjective disturbances found in psychosis. Research on social cognition and mentalizing in psychosis, however, has also pointed to underlying deficits in how persons make sense of their experience of themselves and others. To explore the question of whether metacognitive research in psychosis offers unique insight in the midst of these other two emerging fields, we have offered a review of the constructs and research from each field. Following that summary, we discuss ways in which research on metacognition may be distinguished from research on social cognition and mentalizing in three broad categories: (1) experimental procedures, (2) theoretical advances, and (3) clinical applications or indicated interventions. In terms of its research methods, we will describe how metacognition makes a unique contribution to understanding disturbances in how persons make sense of and interpret their own experiences within the flow of life. We will next discuss how metacognitive research in psychosis uniquely describes an architecture which when compromised – as often occurs in psychosis – results in the loss of persons’ sense of purpose, possibilities, place in the world and cohesiveness of self. Turning to clinical issues, we explore how metacognitive research offers an operational model of the architecture which if repaired or restored should promote the recovery of a coherent sense of self and others in psychosis. Finally, we discuss the concrete implications of this for recovery-oriented treatment for psychosis as well as the need for further research on the commonalities of these approaches.

## Background

Extensive and converging evidence suggests that alterations in the ways persons diagnosed with psychosis understand and think about themselves and others significantly influences the course of their disorder [1–4]. This has fueled research on the processes that might undermine or enhance self-experience [5]. For example, interest has grown in describing and measuring aspects related to self-experience, which if compromised, may contribute to the loss of agency and self-coherence, and if recaptured, may enable recovery from psychosis [6].

One emerging line of research has suggested that the construct of metacognition could quantify the spectrum of activities that, if impaired, might cause the subjective disturbances found in psychosis [7]. This research relies on an integrative model which understands metacognition as a spectrum of activities that includes awareness of specific discrete experiences as well as an understanding of how those specific experiences are connected in one’s broader life experience. This awareness is thought to enable a sense of self and others that impacts momentary experience [6]. This integrated model of metacognition has suggested that reduction in the capacity for metacognitive activity could derail a person’s ability to effectively make sense of and manage the challenges related to psychosis [8]. This work has similarly suggested that the improvement of metacognitive function amid psychosis could enable persons to make personally meaningful sense of their challenges, and then direct their own recovery and pursue a personally meaningful life [9].

While the integrated model of metacognition has generated novel models of the mechanisms of psychosocial treatment for persons with psychosis [10–13], criticisms have been raised concerning whether it is truly distinct from several other constructs related to how persons form ideas about themselves and others. Specifically, it has been asked whether metacognition adds something unique to science beyond what is offered by research on the constructs of mentalizing and social cognition [14–17]. Understanding the extent to which the phenomenon measured in metacognitive research is distinct from the phenomena measured by social cognition and mentalizing research is essential for interpreting findings from these rapidly expanding fields. Such distinctions further ensure that we do not obscure the chances for discovery by studying the same phenomenon with different labels.

To address this question, a comparison of these programs of research in terms of measurement, findings, and applications is needed. When examining whether two research approaches to a similar subject differ, it is expedient to compare their research tools. For example, are the content and psychometric properties of their rating scales, interviews and questionnaires items similar? As will be detailed below, this is challenging in several ways. First, considerable method variance distinguishes measurement approaches for each construct. Second, research into each construct aims to measure self-experience, which involves assessment of complex, fluid and multifaceted cognitive acts which by their nature are difficult to measure objectively and precisely.

To surmount these challenges, this paper will compare research on metacognition, social cognition and mentalizing. After summarizing the constructs, measurement strategies, and general findings across these three areas, we discuss the unique contribution of metacognitive research in terms of experimental procedures, theory and clinical applications. We will suggest how metacognitive research using the integrative model may uniquely describe a set of processes at play as persons make sense of and respond to what is emerging in the world. We will suggest this research reveals a metacognitive framework or architecture which, if compromised, would lead to the loss of persons’ sense of their purposes, possibilities, place in the world and cohesiveness of self, resulting in the subjective disturbances widely observed in psychosis. Turning to clinical issues, we argue that metacognitive research may offer an operational model of the framework which, if repaired or restored, should promote the recovery of a coherent sense of self and others in psychosis. Finally, the concrete implications for recovery-oriented treatment for psychosis will be discussed.

Before beginning, there are several caveats regarding terminology. It has been noted that there is more than one way to understand metacognition in psychosis [18]. To avoid confusion, we are therefore using the term metacognition to refer to a spectrum of activities that are integrative in nature and intersubjectively rooted [7, 19, 20]. Metacognition is integrative which is to say that it enables persons to synthesize or piece together information into a larger whole of themselves or others as unique people. In other words, the whole, which here is a sense of oneself and others as unique persons, is greater than the sum of its parts, which here are individual experiences. To say that these are intersubjective is to say further that the integrating of information into a whole is always done with an actual or imagined other person. For example, any idea formed about oneself is always formed with someone else in dialogue or with an imagined person who could potentially understand and react to that idea. As described elsewhere, we consider this consistent with Flavel’s original definition of metacognition [21], which designated a range of activities that enable reflection and awareness of oneself and others in the world [22]. The other potentially controversial term we have chosen is psychosis. We purposefully chose this term because it is broader than the term schizophrenia while being more specific than serious mental illness, allowing for a flexible application of metacognitive research to real world clinical settings.

## Literature review

### Metacognition and psychosis

#### Conceptualization

The construct of metacognition as studied across cognitive, educational, developmental and abnormal psychology, refers to a set of activities which involve thinking about one’s thinking and responding accordingly to what is happening in the moment in one’s life [21]. The integrative model of metacognition has been applied to explain the alterations in self-experience thought to be at the root of the subjective experience of psychosis. In this model, metacognition has been proposed to refer to a spectrum of activities. These include awareness of discrete experiences which can have embodied, affective and cognitive aspects. This is to say that metacognition can involve being aware of one’s own specific thoughts, beliefs, emotions, desires and experiences within one’s body (e.g. tension in one’s forehead).

Metacognition also makes it possible for a person to see and reflect upon how different specific experiences may be related to one another (e.g. an awareness of how certain thoughts and emotions might be related the experience of bodily tension) and then synthesize those into a broader and evolving sense of oneself and others (e.g. an awareness of how certain thoughts are distressing and lead to tension in the moment within the larger context of a psychological conflict the person has been facing over the last several months) [23]. In this model, metacognition is thus integrative. It involves a process by which individual experiences are understood in terms of their relationship to one another and ultimately integrated into a larger sense of a person as more than the sum of single experiences. The larger sense a person might form of themselves or others, enabled by metacognitive processes, is importantly never static. The holistic sense a person has of themselves is likely to be influenced and changed by new discrete experiences, while the meaning assigned to a specific experience is likely to be influenced by one’s sense of self. For example, an experience of becoming visibly angry might mean something different to someone who thinks about themselves as unflappable and nonreactive as opposed to righteous and enjoying conflict. In parallel, the experience of being visibly angry, if it is remarkable or happens enough times, may lead one to form an idea about themself as different than unflappable and nonreactive.

There are several qualities that metacognition cannot be reduced to in the integrated model. First, the nature of metacognition is not necessarily the product of willful act. A person may decide to reflect upon a given experience or something about the nature of their life and consequently realize something important. Metacognition may also involve automatic processes such that an awareness of a given experience or of something larger going on in one’s life may simply occur to a person without purposefully directing their attention to those issues. Second, metacognitive acts cannot be said to be purely solitary mentalistic acts. A thought about oneself may occur when one is alone. However, regardless of whether another person is present, the ideas we form about ourselves and others are always constructed with a potential audience in mind. For any sense of ourselves we have, there is also a sense of how others, real or imagined, might respond. This is to say that metacognition is not about the formation of final ideas within the solitude of one’s mind but occurs in intersubjective contexts [19]. Third, metacognitive acts are not tied to just thoughts about oneself. A person can notice and form ideas about others, one’s larger community and the possible usages of that knowledge to make sense of and respond to longstanding and newly emergent challenges [7, 20, 24]. Finally, metacognition is not something that can be categorized as intact versus not intact nor compromised by one set of problems. Metacognitive capacity can vary between persons with different degrees of deficits that can be caused by multiple social, psychological and biological processes.

Applied to psychosis, deficits in metacognition have been suggested to result in a sense of oneself and others as relatively more fragmented, resulting in the subjective sense of diminished self-experience reported in qualitative research [6]. In a manner consistent with the initial formulations of Bleuler [25] and Jung [26], decrements in metacognition have been suggested to explain how persons diagnosed with those conditions experience fragmentation of thoughts, affect and will, withdraw from social interaction, and understand themselves and the world in ways that are often inaccessible to others [7].

#### Measurement

To measure decrements in metacognition within psychosis, an interview was developed to elicit a sample of how persons think about their life and its possibilities and challenges. To date, the most commonly used method is a semi-structured interview, the Indiana Psychiatric Illness Interview, which asks participants to offer a general narrative of their lives and experiences of psychosocial challenges [27]. This in turn required the development of a quantitative scale to assess participant’s metacognitive capacity as reflected within that interview. To date, the most commonly used measure is the Metacognition Assessment Scale Abbreviated (MAS-A) [7]. Developed from the Metacognition Assessment Scale [20] (MAS), the MAS-A provides separate scales to assess metacognitive capacity for thinking about the self (self-reflectivity), others (awareness of others), one’s place in the community (decentration) and one’s ability to develop and enact plans based on that information (mastery). The MAS-A departs primarily from the MAS in that the items of each of the four scales reflect a specific metacognitive act that is ordered numerically by level of complexity. Lower scores reflect a more fragmented experiential field per domain whereas higher scores reflect greater degrees of integration. Adequate psychometric properties have been reported [28]. Though less studied, other methods exist for assessing these forms of metacognition including the Metacognitive Assessment Scale Revised [29].

#### Research on psychosocial correlates over the last three years

Using these methods, research has tested the assertion that deficits in metacognitive capacity are prominent elements in a network of disturbances that culminate in the interruption of the lives of persons diagnosed with psychosis. At the level of more basic biological processes, studies have linked metacognitive deficits with alterations in phenomena that underpin social behavior, including levels of oxytocin [30] and linguistic structures that allow for communication [31, 32]. Metacognition has also been linked to interpersonal functioning [33–35] as well as the processes that support social connections, including empathy [36–38]. Deficits in metacognition also have been linked to self-compassion [39, 40] and the richness of personal narrative [41], as well as being implicated in influencing the association between emotional distress and positive symptoms [42]. Other studies have found that alterations in metacognition are linked to changes in neurocognition and, in a formal network analysis, metacognition was found to be a central node in a network that included social cognition, neurocognition and symptoms [43]. Concerning clinical phenomena, a substantial number of cross-cultural studies have linked metacognition with negative symptoms [44–46], in particular with expressive negative symptoms [47, 48], and issues related to clinical insight [8], motivation [49], and intrapsychic function [50].

#### Advances in treatment

As work on links with psychosocial functioning has moved forward, the potential of metacognition as a treatment target has attracted increasing interest. One such treatment is Metacognitive Reflection and Insight Therapy [51] (MERIT). MERIT is a form of integrative individual psychotherapy that seeks to enhance a person’s abilities to form an integrated sense of themselves and others and to use that sense to manage challenges and pursue the possibility within their lives. It conceptualizes the mechanisms of action in a manner analogous to physical therapy. Specifically, patients, within the context of a therapeutic relationship, exercise the ability to form increasingly integrated ideas about the self and others by steadily engaging in metacognitive actions in accord with their current, maximal capacity. Though still in its infancy, research on this approach has suggested that this treatment is acceptable to patients and leads to clinically meaningful gains in randomized trials [52–54]. Qualitative and case studies have linked this approach to metacognitive capacity with growth in senses of agency, historicity, and greater potential for future action [55, 56]. Other work includes a metacognitive oriented social skills training program found to be effective in a randomized trial [57], and interventions focused on the ability to doubt one’s conclusions, which may impact a person’s larger sense of themselves and others [11, 58].

#### Summary

Research generated by the integrated model of metacognition has revealed that many diagnosed with psychosis experience themselves and others in relatively more fragmented ways. These experiences of fragmentation may represent a central node in an interactive network that underlies disability including impairments in other forms of cognition and behavior. It offers a promising model of how both negative symptoms and poor insight may emerge and has spurred the development of novel psychosocial approaches to treatment which may address some of the most subjective and debilitating aspects of psychosis.

### Social cognition and psychosis

#### Conceptualization

Social cognition is a broad term that refers to a collection of mental operations that underlie social interactions, including the perception and interpretation of others’ intentions, dispositions, and behaviors [59]. In research on social cognition in psychosis, most studies have focused on four domains. These include i) theory of mind, or the ability to understand the thoughts and intentions of others; ii) social perception, or the ability to identify socially bound roles and rules; iii) attributional style/bias, or how one infers the causes of events; and iv) emotion processing, or the ability to perceive and use emotions, including identifying emotions in others [17]. Persons with psychosis tend to exhibit deficits in social cognition across all domains [60], and these deficits have been linked to numerous functional outcomes, including a strong connection to community functioning [61]. In addition, these deficits have been found to span the course of the illness, including clinical high risk, early psychosis, and more prolonged groups [62, 63].

#### Measurement

Measurement of social cognitive domains can be broadly categorized into two approaches: social cognitive skills (i.e. the capacity to make correct determinations, e.g. theory of mind) and social cognitive biases (i.e. patterns of interpretation unresponsive to evidence, e.g. hostile attribution bias) [64]. Most commonly, social cognitive domains in each category are assessed with task-based assessments that require a respondent to engage or demonstrate each skill. Self-report and interview-based measures are used less commonly [65], in part because individuals may inaccurately rate their own skill [66]. Measures of social cognition have benefitted from increased emphasis on methodological rigor and reliability through the Social Cognition Psychometric Evaluation study, which aimed to identify measures of the core domains of social cognition and examine their psychometric characteristics [17]. Measures of social cognitive skills had the strongest psychometric support, including one measure of theory of mind, The Hinting Task [67], and two measures of emotion processing, the Bell Lysaker Emotion Recognition Task [68] and the Penn Emotion Recognition Task [69, 70]. To illustrate, the Hinting Task, historically cited as one of the most commonly utilized measures of theory of mind within the field of social cognition [65], is still frequently utilized and tests the capacity for persons to infer the intentions of indirect speech [71, 72]. In this task, participants listen to ten short passages of a verbal exchange between two characters, which concludes with one of the characters dropping a “hint.” Participants must then explain what the character intended by this hint and are scored on the accuracy of their response [67].

#### Research on psychosocial correlates over the last three years

A large body of research has demonstrated the relationship between social cognition and functional outcomes like social functioning, social skills, independent living skills, and community integration [61, 65]. More recent research has continued to corroborate these findings. For example, it has been demonstrated that deficits in thought, language and communication domains are associated with worse functional outcomes, including occupational functioning, ability to communicate effectively in social situations, and general community functioning [73]. Difficulties interpreting verbal cues like humor and emotional prosody [74] as well as visual cues like facial emotion [75] have been linked with poor social communication and functioning in psychosis. Further, deficits in affective prosody recognition have been found to be significantly associated with both role and social functioning in first episode schizophrenia [76]. It has also been demonstrated that affective prosody recognition is negatively correlated with disorganization symptoms, whereas facial emotion recognition is negatively associated with positive, negative, and disorganized symptom domains [76]. More broadly, social cognition, especially domains of theory of mind and emotion recognition, have been linked to elevated disorganization [77], however the relationships between positive and negative symptoms and social cognition are often more complex [78]. Recent results also suggest a role for social cognitive impairments in other meaningful outcomes for people with psychosis, such as suicidal ideation [79] and homelessness [80].

#### Advances in treatment

Work on social cognition in psychosis has led to increased interest in interventions focusing on social cognition. These treatments have generally focused on two methods of intervention: group-based treatments and computerized cognitive remediation. These treatments have shown promise in their impact on social cognition, but study results have been mixed in demonstrating the generalization of clinical benefits to in vivo social functioning. Social Cognition and Interaction Training (SCIT), a manual based group intervention, initially showed promising results with improvements on social cognition measures, including social and emotion perception [81, 82] and social functioning [83]. However, a more recent examination of SCIT found no significant differences between an intervention group and treatment as usual group on several social cognition outcomes, though it should be noted that significant improvements in quality of life, emotion recognition, and social skills were found within the SCIT group [84]. Another group intervention, Social Cognitive Skills Training, has demonstrated positive effects, including improvement in emotional processing [85], social functioning, and psychiatric symptoms [86]. When integrated, cognitive remediation approaches and social cognitive training demonstrate greater improvements in working memory, cognitive flexibility, verbal memory, and theory of mind than a waitlist group, but groups tend to not differ on measures of symptoms and social functioning [87]. These findings have raised questions as to whether improvements in social cognition from treatment may not generalize to long-term or in vivo social functioning. Some have attempted to address this issue by adding an intervention component that allows for practicing social skills; however, this approach appears to have not improved durability of in vivo functioning benefits [88].

#### Summary

Research exploring social cognition has revealed that many diagnosed with psychosis appear relatively less able to correctly identify the thoughts, feelings and intentions of others. Greater levels of these difficulties appear to be a significant source of social dysfunction and are correlated with symptom severity. Social cognitive deficits likely contribute to a vicious cycle in which difficulties correctly apprehending the internal states of others leads to isolation which then leads to exacerbation of other features of psychosis. This work has spurred the development of carefully defined interventions which improve social cognition and can be easily incorporated into existing treatment regimens.

### Mentalizing and psychosis

#### Conceptualization

Mentalizing can be defined as the “ability to understand actions by both other people and oneself in terms of thoughts, feelings, wishes, and desires” [89] (p3). First introduced as a higher-order mental process through which individuals engage in representation and symbolization, the term mentalization was used to characterize the process by which individuals make meaning of human behavior within a context of understanding the mental activities of oneself and other people [90, 91]. Fonagy and colleagues [92] then progressively outlined an attachment-based approach to the development of mentalizing on the basis of observed, early parent-child interactions in borderline personality disorder [93] and other severe mental disorders [94] in which there is a failure to understand oneself because of a lack of access to or a negative regard for others [95]. During this period, the construct of mentalization was relabeled as mentalizing to highlight its procedural character [96] and its explicative and uncertain, imaginative nature [97]. Bateman and Fonagy [89] focused on three non-mentalizing and potentially pathogenic modes: psychic equivalence (i.e. perceiving inner states to be directly equivalent with events in the external world); pretend mode (i.e. decoupling mental life from external reality); and teleological mode (i.e. recognizing mental states only if outcomes are physically observable). Mentalizing was further categorized to include four sub-domains: automatic/explicit, self/other, affect/cognition, and inner-focused/outer-focused [97, 98], illustrating the dynamic nature of mentalizing as nested within varying relational contexts [95]. At any one point, an individual may consider the experiences of others while at the same attending to their own thoughts or feelings. This process may involve explicit, effortful reflection or operate implicitly and prior to any reflection [90].

#### Measurement

Common measures of capacity for mentalizing include survey and transcript-based instruments. The Reflective Functioning Questionnaire [99] and Parent Reflective Functioning Questionnaire [100] are self-report measures designed to assess mentalizing capacity across two subscales, which, respectively, represent salient aspects of mentalizing and evaluate a parent’s ability to understand their child’s behavior in terms of underlying mental states. The Reflective Functioning Scale [101] is a measure used to code mentalizing capacity within an interview that provides opportunities for persons to reflect on their childhood experiences, particularly in the context of their relationships to caregivers (e.g. Adult Attachment Interview). In addition to assessing the patient’s capacity for mentalizing, instruments have been created to assess the therapist’s capacity to mentalize. Such measures include the Therapist Mental Activities Scale, Therapist Relationship Interview, and Mentalization-Based Treatment (MBT) adherence and competence scale, which assess the therapist’s own ability to mentalize as well as their ability to promote the mentalizing process within therapy [91]. A final approach to measuring mentalizing, the interactional approach, derives from the assumption that mentalizing occurs in relationships with others, or within intersubjective contexts [91]. An example of this is Conversational Analysis [102], a qualitative method that examines the structure and process of social interactions, such as a therapy session. Across these studies, it may be noted, the non-mentalizing modes (i.e. psychic equivalence, pretend mode, teleological mode) are only partially and indirectly assessed.

#### Research on psychosocial correlates over the last three years

Deficits in mentalizing have been tied to a multitude of poor outcomes [103]. Research has found that deficits in mentalizing are linked to decreased community engagement, reduced vocational functioning, as well as poorer quality of life [90, 104]. Other studies, which have investigated whether aggression and violence are linked with impaired mentalizing, offer conflicting views. One study found that psychotically driven aggression was linked with poorer mentalizing capacity [105], while another found no difference in mentalizing between individuals with schizophrenia spectrum disorders who are violent versus those who are non-violent [106]. A recent study found that mentalizing deficits act as a mediator between childhood abuse and negative symptoms in that the effect of childhood abuse on the severity of negative symptoms was amplified by the absence of the ability to mentalize [107]. Most generally, a recent study conducted by Boldrini and colleagues [108] suggests that the presence of impaired mentalizing may predict the development of psychosis itself.

#### Advances in treatment

Inspired by research on mentalizing and the emerging recovery movement for individuals with psychosis, psychotherapeutic interventions have been developed to target mentalizing deficits in psychosis [90]. Mentalization-Based Treatment for psychosis (MBT-p) is an adaptation of a therapeutic intervention originally developed for borderline personality disorder. The primary premise of MBT-p is that psychosis entails core deficits in the areas of awareness of one’s own mind (e.g. failing to differentiate between one’s thoughts and external stimuli) and the minds of others (e.g. difficulty in interpreting others’ intentions) [109]. As a dynamic practice, MBT-p aims to promote mentalizing ability to strengthen the ability to make sense of interpersonal experiences and to better navigate social life by recognizing how various social situations affect thoughts, feelings, and motivations [90]. Interventions focus on the patient’s experience in the present through the therapist’s continued curiosity about the patient’s thoughts, feelings, and intentions as well as by encouraging the sharing of narratives of social interactions [103]. Case studies have reported that MBT-p is linked to improvements in social functioning [103] and the decrease of negative symptoms. A recent, randomized-controlled trial found MBT-p led to significant improvements in social functioning at post-treatment and at 6-month follow up. Results further revealed a trend toward better performance on measures of mentalizing ability in the MBT-p group and an increased effectiveness of MBT-p in early psychosis versus prolonged psychosis [110].

#### Summary

Research exploring mentalizing has revealed that many diagnosed with psychosis appear to experience less secure levels of attachment and as a result are relatively less able to consider the experience of others while attending to their own thoughts or feelings. This process may lead to reduction in persons forming coherent ideas about what others want, think and feel. As has been found in social cognition, greater levels of these difficulties appear to be a significant source of social dysfunction. This work has also spurred the development of interventions with psychodynamic roots which are less structured than social cognition interventions and focus on noticing and developing the ability to think about others and one’s own relationship with them.

## The unique experimental, theoretical and clinical implications of metacognitive research in psychosis

As illustrated in literature reviewed above, research on metacognition in psychosis shares in common with research on social cognition and mentalizing a concern with problems persons have making sense of their self-experience and the experience of others. Metacognition is concerned with the degree to which persons sense of self and others is integrated as opposed to fragmented. Social cognition is concerned largely with the degree to which other people’s emotional and cognitive experiences can be correctly recognized and understood. Mentalization is concerned with the formation of ideas about oneself and others in the context of a history of differing kinds of attachments with others.

Given these commonalities, as noted at the outset of this paper, one question that arises is whether metacognitive research in psychosis is really exploring anything distinctive relative to research pursuing social cognition and mentalizing. In response, we would suggest that this review considered as whole reveals several unique advances offered by metacognitive research in terms of experimental procedures, theory and clinical information that warrant its continued status as a unique construct.

### Distinctive experimental procedures

First, metacognition, as operationalized using the MAS-A, diverges from social cognition and mentalizing research in its emphasis, consistent with work on personality disorders [24, 111–113], that the senses of self and others that are disturbed in psychosis involve automatic and purposeful processes in which embodied, affective and cognitive experiences are not successfully integrated into a larger sense of the self or others. Metacognitive research thus distinctively resists framing the experience of the self and others as fundamentally modular or the product of different dissociable processes. As something that is more of a multidimensional activity than an aggregate of discrete states, metacognition allows for an awareness that is larger and more complex than the sum of any supposed parts. It describes the processes though which persons not only experience the world but make interpretations of those experiences and adjust both their behavior and ideas about that experience over time in ways that can range along a continuum of fragmentation to integration.

In contrast to social cognition research, metacognition is operationalized in a way that allows it to go beyond measurements of the correctness of a certain perception. The central tool of this research, the MAS-A, offers a means of measuring processes that allow a person to be more or less able to continually interpret and adjust their sense of who they are, how they are related to others and what they are doing in the world. Its four dimensions allow for an assessment of the degree to which persons with psychosis are fitting together information in an ongoing manner as they make sense of and respond to what is happing in their lives in terms of their well-being and needs, even during periods of significant confusion and distress. A metacognitive deficit is thus distinct from a social cognitive deficit. Whereas a social cognitive deficit results in an identified point of failure (e.g. mistaking someone’s emotional state), a metacognitive deficit leads to failures in the process of evaluating and responding to a changing world given one’s own values or history. A metacognitive deficit thus does not represent an error, but instead less efficacious activity within a network that is integrating information across time and using that integrated information in response to shifting contexts. It accordingly has the potential to provide a unique and measurable marker in the puzzle of how disturbances in genetics and basic brain function and forms of social injustice are linked to the struggles of a particular person seeking to move forward in their lives in the midst of psychosis.

In common with mentalizing research, metacognitive research understands intersubjectivity as a basis for the mutual understanding which underpins interpersonal relationships. Metacognitive research distinguishes itself from that approach, however, in its consideration of the role of each person’s unique interpretation of and response to what happens when action is taken on the basis of those interpretations. As operationalized by the MAS-A, metacognition allows for the measurement of a more multifaceted kind of meaning making, one in which there may be more complex interactions than afforded by most approaches to measuring mentalizing. By considering through the use of separate scales on the MAS-A both the ability to form ideas about oneself and others in the larger world (i.e. decentration) and use reflective knowledge to respond to psychosocial challenges (i.e. mastery) as distinct from self-reflection and reflection about others, the metacognitive approach offers more points of access to disturbances in subjective experience in psychosis, which may be why measures of metacognition tend to be more closely associated with measures of psychopathology than measures of mentalizing [7, 114]. Finally, metacognitive deficits also appear to have consequences or manifestations in the world distinct from those of mentalizing deficits. When viewed from a broad perspective, metacognitive deficits culminate in the loss of an ability to fully engage in and experience membership in one’s community through shared participation in its activities while accepting and fulfilling required roles and holding certain values within that community. In contrast, with mentalizing deficits we see changes that are more focused on the distress of an individual person, including the loss of emotional equilibrium, internal balance and comfort with key social attachments.

### Distinctive theoretical aspects

As metacognitive research has enabled the measurement of processes that support a persons’ making sense of and responding to experience, and then continually readjusting, it also offers ways to think about the person who is experiencing psychosis. Specifically, beyond the contributions of the literature of social cognition and mentalizing research, metacognitive research offers a framework to think about the person who is living with psychosis and not just their errors or discomfort. It allows us to understand particular ways in which the self is experienced which may offer a unifying conceptualization of more than a century of observations about the disturbances in the phenomenology of psychosis.

Since the forebearers of existential philosophy, including Kierkegaard [115] and Nietzsche [116], self-experience has been understood as dynamic and multifaceted, and as something experienced as taking place in changing, diverse situations of a person’s life over time. This is to say, consistent with the experimental methods of metacognitive research, that persons’ experience of themselves involves an ongoing process in which they interpret and respond to what is happening to them in the moment and over larger periods of time [117]. As described in the early stages of modern psychology [118], we experience ourselves as we participate in the world, and so self-experience is always a function of an interaction [119] and something that is dependent upon a given context [120].

In this light, beyond what is offered by social cognition and mentalizing research, metacognitive research helps us understand how disturbances in self-experience emerge in psychosis as the interactions and integration which allow for a sense of self are compromised by metacognitive deficits. It is not the *what* of a particular perception (as in social cognition) but the *how* of the unique way in which an individual organizes their understanding of their life.

Specifically, we suggest that research on metacognition in psychosis allows us to see how disturbances in four interrelated but conceptually distinct dimensions of self-experience: purpose, possibility, positionality, and partiality could emerge in psychosis and affect persons in the ways revealed in empirical research [121]. The first two, *purpose* and *possibility,* refer to how persons know themselves relative to the formation of an ongoing sense of the aims they are pursuing in their lives and what may possibly come from that pursuit. These purposes, which distinguish a person as a unique being, can be immediate, for example, accomplishing a particular task, or larger in the sense of becoming a particular type of person. The possibilities at hand can involve a sense of the means or the barriers to achieving a potential end.

With the deficits in metacognition found in psychosis, however, a person might experience themselves as without a purpose or hope for fulfillment ahead of them. For example, impairments in self-reflectivity could concretely weaken the links between different experiences, disabling the potential to recognize one’s purposes and possibilities in the moment and their evolution over time. A person might make a correct judgement about a discrete experience but be unable to see the web of relationships that exist in the moment and as they evolve over time give it a particular meaning. The range of purposes that could be pursued in the flow of life would, therefore, be reduced as would the ability to assess how well one is achieving those purposes. Moreover, one would be less able to evaluate whether those purposes remain worth pursuing. And without a reply to questions like, “why am I doing something” or “is it worth doing this?”, it would be very difficult to answer the question, “who am I?”

Matching observations from psychiatric rehabilitation of alterations in self-experience in psychosis, a person might appear to have lost a sense of why they should do something they had been doing for years as well as what might come from doing something that is meaningful [122]. This is also consistent with narrative research, which has found that a sense of purpose is less discernable in the life stories of persons with psychosis [41, 123, 124]. More generally, without a sense of one’s purposes and possibilities to anchor a growing sense of self, the symbolization of self and experience might diminish as found in psychoanalytic accounts of the subjective experience of psychosis [125–127] and match what has been described as a lack of existential agency [128]. Finally, without purposes emerging in response to and alongside possibilities, and thus without a working sense of who one is, a sense of the uniqueness of one’s experience might also wane, leading to states described by phenomenologists of self-experience in psychosis as minimal and lacking the quality of *mineness,* or what is referred to as ipseity [129, 130].

The third kind of disturbance that could affect sense of self in psychosis could occur in one’s sense of *position*, or how the self is experienced as contextually related to others, social structures, or a point in history. For example, in a given moment, the sense one has of oneself, including one’s purposes and possibilities, may involve seeing oneself as a current teacher and former student, a former lover but longstanding friend, or a current detractor of someone one supported in one’s youth, which one now regards as overly conservative. The possibilities and purposes that are inexorably part of a sense of self are not free-standing or abstract ideas but accrue meaning (and only become possible) because they are in relationship to other people in multiple contexts. With the kinds of metacognitive deficits observed in psychosis, especially decrements in one’s awareness of others and broader community, past connections linking one’s immediate experience to those of others would also become significantly less available, diminishing the sense of one’s position in the world and possibilities regarding who one could become. If the capacity for mastery, or the ability to use reflective metacognitive knowledge to respond to psychosocial problems, were impaired, persons would be left with little sense of how to manage their lives, diminishing their sense of possibilities, including cooperative action with others, which would further erode a sense of who one is relative to others in the world.

Corresponding to experiences described by earlier psychoanalysis [131, 132] and existential psychiatry [133, 134], this would result in a person with psychosis experiencing interactions with others as fundamentally disturbing and relatively lacking a sense of intersubjectivity. Instead of an ordinary interaction offering reassurance about one’s place in the world, it might only provoke more confusion and hence increase feelings of vulnerability and worries about the intentions of that other person. This is also consistent with descriptions of the devastating loss of one’s place in the larger community as described by persons with lived experience of psychosis [135] and matches accounts of the centrality of recapturing that place in recovery [2, 136].

The fourth kind of disturbance in self-experience that may emerge in the wake of metacognitive deficits in psychosis could be referred to as a lack of *partiality*. Partiality is intended to refer to an ongoing sense that the self can never be captured or characterized by any one thing and instead is made up of many things that are likely to change. In other words, a healthy sense of self is partial in that it cannot be reduced in a lasting final form as defined by one set of wishes, thoughts, or emotions. With the deficits in self-reflectivity and decentration found in psychosis, persons would be unlikely to have a working sense of themselves as having multiple facets which may be contradictory and change over time. Without this quality of partiality, the cohesion of a larger sense of self would be threatened by inconsistencies and dissolve [137, 138]. This could result in a sense of self in which different aspects of the self are experienced as active but unrelated to one another, where no aspect of the self is distinguished from another, and hence all are experienced as absent.

This is not to say that social cognition and mentalizing are not involved in a unique person’s purposes, possibilities, positions, and partiality. They are. With deficits in social cognition, it would be more difficult to discern one’s positions, especially as they pertain to interpersonal relationships, and that would compromise one’s ability to understand and pursue intimate relationships and to cooperate with others. Similarly, it would be difficult for a person to describe and pursue larger purposes in life if one’s mentalizing abilities were weakened. Yet neither view quite captures the temporal dynamism nor the breadth of the dimensions that can be quantified and mapped by the integrated, metacognitive perspective. Though social cognition is able to clearly describe and measure discrete elements of experience, it cannot capture subjective, unique features of an individual’s experience of themselves (e.g. in terms of one’s purposes, possibilities, positions, and partiality). Similarly, mentalizing clarifies connections between attachment, affect and the forming of a sense of others, but it also is not able as comprehensively as the metacognitive approach to consider the broader range of activities and interactions that make up community participation and the experience of one’s life as uniquely unfolding. Allowing an understanding of how someone makes sense of the suffering inherent in their experience of psychosis, metacognitive research uniquely allows us to understand and relate to the suffering of different persons in terms of their experience of everyday life.

### Distinctive clinical applications

Practical issues of treatment reveal perhaps the most distinctive features of the research on the integrative model of metacognition. If metacognition describes an architecture or framework for meaning making, which if compromised results in profound subjective disturbances, then it may also describe an architecture which if repaired or reconstituted should promote recovery along these same subjective lines of experience. One clear implication is that treatment requires more than addressing specific deficits or remediating impairments in specific skills. It may require the promotion of the ongoing integration of experiences. This integration would be expected to enable persons to recapture a sense of self-cohesion which includes a rich sense of what they are seeking in life, what is possible for them, and where there is a meaningful place for them in the world. It would also be expected to cultivate an ability to acknowledge and creatively respond to experiences which offer any number of significant challenges or possibilities.

This is not to say that social cognitive approaches might not also enable persons to make sense of what they are facing. It’s direct effects though seem limited to helping persons make fewer errors. With fewer errors or greater awareness of how one reasons about others, a person might well be able to make more complex kinds of meaning later, but that later process is left up to the patient to carry out elsewhere. Certainly, mentalization treatments in common with the metacognitive approaches noted above, including MERIT, understand intersubjectivity as a requirement for psychotherapy to effectively address how persons think about themselves and others. Yet some metacognitive approaches to treatments, such as MERIT, also factor in each person’s unique interpretation of and response to what happens when those interpretations are enacted while also emphasizing how disturbances in or support for one’s sense of self come from multiple sources outside of dyadic exchanges. By considering decentration as its own, quantifiable variable, metacognitive treatment approaches may have broader opportunities for understanding outcomes resulting from these interventions.

## Conclusions and further directions

In this paper we have sought to address whether research employing the integrated model of metacognition offers unique insights in psychosis relative to social cognition and mentalizing research. To that end, we have presented the key conceptual features of and recent research from each field of study, all of which point to disturbances in the experience of the self and other. We then offered a review of four aspects of self-experience relevant in psychosis about which metacognitive research may offer unique insights. On the basis of this, we have suggested that metacognitive research brings added breadth and dynamism to the study of self-experience in psychosis. It uniquely reveals a dynamic architecture that, should it be compromised, may result in specific disturbances in persons’ sense of themselves in terms of their purposes, possibilities, position in the world and cohesion in the face of contradiction. From this vantage point, we see a broader interactive, socially situated network of capacities in which persons encounter and respond to challenges as members of a community, which affords a uniquely rich sense of what promotes and what blocks the path to recovery.

Practically, metacognitive research illuminates how subjective disturbances in psychosis can be reflective of more than a singular failure at one focal point or state of emotional distress, leading to a vision of treatment that returns to fundamentally grappling with issues of meaning making and agency. Metacognitive research thus makes it possible to begin to think of psychotherapy that addresses metacognition as a “technology of the self,” in the sense offered by Foucault [139]. In addition to teaching skills and addressing symptoms, this research reveals how treatment can also engage persons with psychosis in the construction of meaning and community membership, which includes noticing what has happened to them, what they need, as well as what is potentially just and unjust in their community.

There are limitations, however. We have focused on the singular contributions of one model of metacognitive research in psychosis. It would be fruitful to consider other measurements of metacognitive capacity and analyze them in a similar light. Moreover, more work is needed to determine whether unique contributions also can be found in the research tracking social cognition and mentalizing across disturbances in the experience of self and others in psychosis. Other work is needed that measures all three constructs and their unique and cumulative effects on outcome. While epistemological differences and implications can be discussed, the differences in quantitative studies are a direct function of the validity and reliability of the reported tools. Some of the results on which we based our hypotheses may therefore reflect measurement biases rather than real theoretical and clinical differences.

Finally, we have not considered the overlap between mentalizing and social cognition which in some work have been considered as overlapping themselves [140]. We have not explicitly explored the overlap or commonalities among metacognition, social cognition and mentalizing research in psychosis. Despite the differences revealed here, there are elements common to all three and to various pairs. Future work is needed to explore these commonalities in order to work towards a unified model of subjective disturbance in psychosis and the requirements of recovery-oriented treatments.

## Data Availability

Not applicable.

## Abbreviations

MAS-A: Metacognition Assessment Scale Abbreviated
MAS: Metacognition Assessment Scale
MBT: Mentalization-Based Treatment
MBT-P: Mentalization-Based Treatment for Psychosis
MERIT: Metacognitive Insight and Reflection Therapy
SCIT: Social Cognition and Interaction Training
